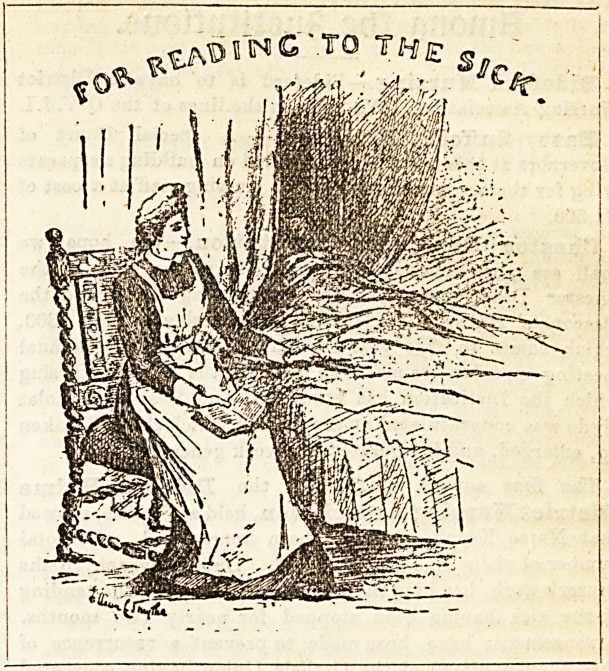# The Hospital Nursing Supplement

**Published:** 1892-04-23

**Authors:** 


### "The Hospital" Nursing Mirror.

Being the Extra Nursing Supplement of "The Hospital" Newspaper.

Contributions for this Supplement should be addressed to the Editor, The Hospital, 140, Strand, London, W.C., and should have the word "Nursing" plainly written in left-hand top corner of the envelope.

### En Passant.

THE ENDOWED BED.—Our subscribers who came forward so willingly at our appeal for the funds of this bed will be glad to hear that three nurses have already benefited by it, and a fourth is just going to stay at the Home, so the need of its existence is fully proved. We shall be glad if in future any kind subscribers will send their donations to Editor, *Nursing*, at this office, and applications for the use of the bed had better be sent to Miss Holditch, The Brassey Holiday Home, St. Leonards-on-Sea. Miss Holditch and Sister Frost have had 529 visitors at the Home. The institution is just now entering the third year of its existence.

PROBATIONERS' "TIME OFF DUTY."—Most hospitals have a printed set of rules, also a paper giving the hours of the nurses "on" and "off" duty, and these are generally put into the hands of applicants who wish to be trained. There are trifling variations in the papers of different hospitals, but there are graver discoveries to be made as to the carrying out of these rules. For instance, in one case the daily "two hours off duty" means literally and honestly, two hours in which the probationer is free from all duties and responsibilities, whilst in another institution the young nurse soon learns that on one day in the week a class for theoretical teaching has to be attended during this period, and on a second afternoon her "time off" is also partially disposed of for her. At yet another hospital the hours are represented according to the exact time which the probationer can spend out of doors, and no mention is made of the extra fifteen minutes given for removing and for donning her out-door garb. In fact, whilst some "time tables" are better than they seem, others sound better than they are. We, therefore, advise all would-be nurses to enquire from the first exactly what privileges they will be entitled to claim, as they will thus avoid many little disappointments afterwards.

"A HOME FOR THE DYING."—Our readers will remember the mention of this Home last October, or rather the need of more accommodation for those who are just entering the valley of the shadow of death. Gladly we learn that already a suitable house has been found at "Sunnyside Swiss Cottage;" it stands in two acres of ground, and will hold forty to fifty patients with comfort. The house has been paid for, but the Committee now await further donations to enable them to make the necessary alterations, and to buy furniture with. To be penniless and dying, and to have no place to lie down and die in—can anything be more pathetic? "But there is always the workhouse," thinks the practical reader. Yes, so there is, and where the skilled hands and kindly hearts of the Workhouse Infirmary Association nurses are found tending our dying poor we know the way is made as easy as possible for them. But, unfortunately, in spite of the ever-increasing number of lady Guardians and the general enlightenment of Boards of Guardians, these servants of the sick are not found in all workhouse infirmaries, all shame to the public! and so the room and care for the dying must be supplied elsewhere. "Sunnyside" will be open for inspection on Saturdays from three to five, and reports of last year's work can be obtained from Miss Davidson at the old home, 5, Oakhill Park, Hampstead.

LINCOLN NURSES.—By mistake last week we mentioned the receipt of the *fifth* annual report of the Lincoln Institution for Nurses; it should have been the *twenty-fifth*.

MIDWIVES' REGISTRATION.—On the motion of Mr. Fell Pease, the Select Committee on Midwives' Registration was nominated as follows: Mr. Bright, Mr. Tatton Egerton, Dr. Farquharson, Sir F. Fitz Wygram, Dr. Fox, Mr. Howorth, Sir Guyer Hunter, Mr. Fell Pease, Mr. Rathbone, Mr. Stephens, and Mr. Arthur Williams; five to be the quorum.

THE "HOUSE."—If some of the poor creatures who have suffered so sadly and amidst such squalid surroundings this winter, and who have scorned the suggestions of the parish doctor and district visitor that they "would be better in the infirmary," could obtain a glimpse into the wards of these institution they would be of a different opinion when next overtaken by sickness. A visit to Marylebone Infirmary at Notting Hill gives a wonderfully agreeable impression. The white beds which contain the silver-haired old women, with their snowy caps and scarlet shawls, are not suggestive in any way of that badge of pauperism which is painfully conveyed by the outdoor costumes of most workhouses. But Marylebone is an exceptionally favoured institution, for it is also a training school for nurses on the Nightingale system, and it has a fine nurses' home, supervised by a fully-trained Sister, and the Matron is well known in nursing circles for her all-round abilities, as well as for her unusual power of organisation.

PROGRESS OF DISTRICT NURSING.—Want of space has prevented us giving an account before now of the formation of a District Nursing Association for Aberdeen, to be affiliated with the Edinburgh Committee of the Q.V.J. Institution. Miss K. M. Lumden, of Aberdeen Hospital for Sick Children; and Miss R. F. Lumsden, of Aberdeen Royal Infirmary, are working hard to set the Association going, so we may look to speedy success. The Lord Provost Stewart took the chair at the crowded meeting in the Music Hall building, and the "fors and againsts" affiliation were thoroughly thrashed out, the very obvious advantages of becoming Queen's Nurses being agreed to by all speakers. It seems extraordinary that there should ever be much divergence on this point. At Aberdeen the advantages of affiliation will be numerous. Number one would be that the services of a thoroughly trained district nurse would be at once obtainable; they would be able to claim help from the Aberdeenshire Branch of the Scottish Needlework Guild, and, what is most conclusive, they would work in unison with the whole of the rest of Scotland. At Glasgow, where district nursing was started long before the Q.V.J.I. was founded, the Association has, for the sake of unity, affiliated to Edinburgh. The trustees of the late Mr. R. Ronaldson, who left funds seventeen years ago to help to provide nurses for the poor, are going to aid the new Association with the money at their disposal. Whether the scheme will be worked with the Aberdeen General Dispensary, the Royal Infirmary, or the Children's Hospital remains to be seen, but it is absolutely refreshing to find how largely the Aberdeen folk realise the power of co-operation in good deeds, and the saving that is to be done in administrative expenditure. The Committee now in process of formation is authorised to collect funds, and Miss Laing, 5, St. Swithin Street, who has consented to act as Treasurer, will receive contributions. All success to Aberdeen!

### Ventilation, Disinfection, and Diet.

By P. Caldwell Smith, M.D.

*(Continued.)*

Vitiation of Air—Combustion of Coal and Coal Gas—Composition of Coal Gas—The best Light for Sick Rooms—Amount of Fresh Air Required for Each Person—Standard Purity of the Air—Amount Required for Lights.

The air of inhabited rooms may also be vitiated by c.mbustion. When coal is burned, carbonic acid, carbonic oxide, sulphuretted hydrogen are produced, but the more perfect the combustion the less carbonic oxide and sulphuretted hydrogen is produced. One pound of coal requires as much as 240 cubic feet of air for complete combustion, while one pound of wood requires only half that amount. Wood also produces little sulphur in its combustion.

Sanitarily speaking, the most important impurity produced in the combustion of coal is carbonic oxide, this being more liable to be produced when the combustion is incomplete. It is, however, most commonly produced from the combustion either of coal or coke in iron stoves, although it may also proceed from imperfect combustion of ordinary coal gas. The fuel which most readily produces CO is anthracite, or as it is called in Scotland, blind coal or coke. This produces CO, as already stated, when there is not an abundant supply of fresh air, but we can never say that a coke or anthracite fire produces no CO. "In a bed of live coals, in the centre of the fire, we know that it is present in large amount because there is a deficiency of O; it then passes through the upper layer meeting with more O, and becoming further oxidised or burnt, making a bluish or yellowish flame, and forming $CO_2$." CO is a most violent poison, even in very small quantities, producing a sense of general oppression, weight about the head, and difficulty of breathing. This question of production of CO in cast-iron stoves is very important, as it shows that no room should be used as a sick-room which is heated by one of these. If it is unavoidable that a stove must be used, then it should be of wrought iron, not of cast iron, and it should also be lined with fire-clay brick; the reason for this is that it has been proved conclusively that the CO passes through the pores in the cast iron, while it passes only to a very small extent through wrought iron.

It is necessary to say a little at this stage regarding the pollution of the air of inhabited rooms by coal gas, as in nearly all houses this is the only illuminating agent used. The amount of vitiation of the air depends to a very large extent on the purity of the gas. Coal gas is by no means a simple product. Its main component is hydrogen, but it also contains marsh gas, carbonic oxide, carbonic acid, nitrogen, and several hydro-carbons, these, as benzene, &c., being the principal illuminants. When it is burnt the products of combustion escape into the rooms, these products being nitrogen, carbonic acid, carbonic oxide, with sulphurous acid and ammonia. The public are now to some extent beginning to recognise the necessity for having ventilated gas jets, in which all these products pass at once into the open air. In some forms of ventilating gas jets, not only do these products pass out, but the air for the combustion of the gas is also taken from the outside directly, thus necessitating the introduction by means of ventilation of a smaller amount of pure air than would be necessary if such were not the case.

It is apparent from what I have said that the external air must necessarily (and more especially is this the case in manufacturing towns and cities) contain a large amount of these products of combustion, and if it were not for constant diffusion and other processes of nature, as rain, wind, &c., the air would soon get poisonous. In rooms this holds good to a much greater extent. The atmosphere is not only polluted with $CO_2$ and organic matter from human beings, but also by all the above products of combustion both of coal and coal gas.

In a sick-room, then, I am frequently asked whether should an oil-lamp or gas be used. If light is not the first object, then I should unhesitatingly say a good, well-trimmed lamp is best, and for these reasons: An ordinary gas-burner burns about three cubic feet of gas per hour, producing six cubic feet of $CO_2$ and consuming all the O in 24 cubic feet of air. An ordinary lamp, not, however, giving the same amount of light, burning 154 grains of oil per hour, consumes the O of only 3·2 cubic feet, and produces only a half cubic foot of $CO_2$.

The products of combustion of oil are hardly so deleterious as those of gas, although if a bad lamp is used a certain amount of a peculiar organic vapour is given off, which, though not proven to be injurious to health, is still decidedly unpleasant to the smell. There are no compounds of sulphur given off from oil, while these are given off in large quantity from gas. In Glasgow and the neighbourhood alone it has been estimated that the coal consumed gives off sulphur compounds equivalent to 300,000 tons of oil of vitriol or sulphuric acid annually. The external air may be vitiated in other ways, as by the emanations from ashpits, sewers, and cesspools, these being, especially in a concentrated form, extremely unhealthy.

By ventilation, then, as we have seen, is meant the removal or dilution by pure air of the skin and lung exhalations and of the products of combustion in inhabited rooms. We have first to know the amount of fresh air that is required by each individual. Each person requires three to four cubic feet of air per minute, while, as we have already seen, a man at rest gives out on an average ·6 of a cubic foot of carbonic acid.

What, then, is to be considered the standard purity of the air in inhabited rooms? This is pretty easily remembered, as it is the same figure as amount of $CO_2$ given off, viz., ·6 per 1,000 volumes, ·4 per 1,000 being the normal amount of $CO_2$ in the air, while ·2 is what is called respiratory impurity, or that due to exhalation from human beings and the products of combustion.

To find the number of persons who may occupy a room involves only a very slight exercise of the simple rules of arithmetic: height × breadth × length. The number of cubic feet of air required for each person is a variable quantity. In the London lodging-houses 300 cubic feet is the required amount, while in the new Glasgow Police Act 400 is insisted on. Soldiers are allowed 600 cubic feet, while in hospitals the amount varies from 1,200 to 2,000 cubic feet, and in infectious disease or fever hospitals much more is necessary. In ordinary bed-rooms 600 should at least be insisted on, while if a room is used as a sick-room, double that amount is an absolute necessity. Any ordinary sized bed-room will contain the requisite number of cubic feet. It must also be remembered that the larger the room the less frequently has the air to be changed, and the fewer draughts will be produced.

The amount of fresh air requisite for each individual is as follows:—For adult males, 3,500 cubic feet per hour; for adult females, 3,000 cubic feet per hour; for children, 2,000 cubic feet per hour; average 3,000.

The advantages, for ventilating purposes, of securing a fairly large room for a sick-room may be seen more definitely if we take an example. Take a room of 300 cubic feet, there the air has to be changed ten times in one hour if 3,000 cubic feet are to be supplied, while in a room of 1,000 cubic feet, three times an hour only is necessary for the same amount of ventilation. It must also be distinctly remembered that the largest space can only provide air for a certain time, and when the limit of respiratory impurity is reached the same amount of fresh air has to be supplied.

Besides the 3,000 cubic feet per hour necessary for each individual, the amount required for lights has also to be considered, and as an ordinary burner burns three cubic feet of gas per hour, and each cubic foot of gas requires 1,800 cubic feet of air for dilution of the product of combustion, 5,400 cubic feet extra must be supplied during the time the gas is lighted.

### Male Nurses.

*(From a Correspondent.)*

Nursing on scientific and hygienic principles is now recognised as one of the necessities of the times, and if we contrast the present system with the antiquated one in vogue some few years since, we can only feel surprised that the old state of things should have been allowed to continue for so long a period.

Female trained nurses have established themselves as a regular and honourable body of professionals, and to many of their poor suffering patients have proved to be "ministering angels" in their noble work.

It is the object of this paper to advocate the cause of an increasing and, we hope, as honourable a body, viz., the male trained nurses. As yet the male trained nurses labour under many difficulties—the need of their services is not fully recognised—but there are many cases which necessitate the employment of male attendants, and in such cases when a trained attendant is not employed, how often do the patients suffer annoyance and inconvenience from the well-intentioned but mistaken efforts of relatives and friends.

Without wishing to detract in the slightest degree from the capacity and gifts of our sister nurses, yet there are many circumstances which combine to make trained male nurses more capable of aiding their brothers in distress. In epileptic cases the violent spasms require the powerful restraint which only a man can exercise, and it often happens that a strong "animal," who lacks any knowledge, is called in and the poor sufferer is treated in an almost inhuman manner. Also the heavy weight of most men requires more lifting power than females are capable of, and there are many other obvious reasons why the male nurse has now become a necessity. In what way then is publicity to be given to the movement, and what means adopted to ensure its success?

In the first place it is necessary that those who devote themselves to the work should be heart and soul in it and be physically and mentally trained for the occupation, and it should be a *sine qua non* with the heads of nursing establishments that their employés should only be chosen from among fully qualified men. There is a great objection to the existing custom of sending out nurses indiscriminately, those who are specially qualified by experience and education for certain cases should, whenever possible, be chosen to deal with them, otherwise the injury inflicted, not only on the institution concerned, but on the whole body of male nurses, by one mistake in this direction, will be great and far reaching; while, on the other hand, by sending out nurses thoroughly acquainted with their subject, confidence is inspired in the patient and the work of the medical man is materially assisted.

The look of relief may often be seen on the face of the harassed practitioner when he finds that he has a skilled nurse, in whose hands he can safely leave his patient, assured that his directions will be carefully carried out and a faithful report of the case given on his next visit. The medical profession welcome us heartily, and we, for our part, must show ourselves worthy of their confidence. The issue rests principally in our own hands, and if each member of the nursing profession is impressed with the nobility of his calling and enthusiasm for its success, the object we have in view will be speedily attained.

### For Reading to the Sick.

### THE VINE.

Have we ever noticed the difference between a vine that has been carefully trained and pruned and one that has been neglected for years? The latter is full of leaves, and runs riot just as it wills, catching with its tendrils on anything which comes in its way; here making an old building picturesque with its graceful festoons, and there garlanding a rugged tree "with leaf and berry and flower through and through." It looks very pretty, but what about the fruit when thus uncultivated. The bunches are few, the berries small, and their flavour poor and acid. All its strength and vigour has been spent in aimless growth and wandering, in escaping the vine-dresser it has become worthless. On the other hand a vine which is cultivated may not be so picturesque to the eye of the casual observer, but there is a certain charm and satisfaction always in the uniformity with which it is trained. Only a certain number of shoots are allowed on each branch, and these go perfectly straight upwards, always upwards, and are nailed tightly back, while every useless leaf is broken off, with the result that the grapes are large, sweet, and luscious, and hang in rich profusion in the master's house. Now the use of a vine is to bear fruit, the wood is of no importance for any other purpose, nothing can be made of its hollow, tough stems, they are simply constructed to carry the sap which rises from the root and supplies all the nourishment.

We may learn a valuable lesson from the vine if we read and ponder on the 15th chapter of St. John's Gospel. There our dear Lord compares Himself and His disciples to the vine. He says, "I am the True Vine, and My Father is the husbandman, every branch in Me that beareth not fruit He taketh away, and every branch that beareth fruit He purgeth it that it may bring forth more fruit." This is a warning to us to try and bear fruit, and it also makes clear why we are afflicted in our bodies, why our heads ache, why our limbs contract with pain; it is to prevent our running wild, and to make us bear more or better fruit, worthy the root from which we spring. It is irksome to be brought into such strict order as a good vine requires, naturally we like to be our own masters and do as we will. To have our leaves nipped off, that is, our pleasures lessened; to be nailed to a wall, as it were, by a broken limb or an attack of fever, these things go very much against the grain. But we must not kick against them, they are not sent as punishments but to clear off our vagrant shoots and superfluous leaves so that we may bring forth beautiful clusters of the fruits of the Holy Spirit—love, joy, peace, long-suffering, gentleness, goodness, faith, meekness, temperance. What a splendid bunch of virtues! What a glorious imitation of the Lord, who was Himself perfect in every good word and work! Ours then let it be to draw our nourishment from the True Vine, and to abide in Him ever for our strength and vigour.

### Among the Institutions.

**Bideford Nursing.**—Bideford is to have a District Nursing Association. We hope on the lines of the Q.V.J.I.

**East Suffolk Hospital.** — A special Court of Governors at this hospital has decided on building a separate wing for the accommodation of the nursing staff at a cost of £1,500.

**Chester Deaconess Institution.**—We hope we shall see more subscriptions coming in to the work of the Chester Deaconesses, both for the Nursing Home and the Deaconess' House. The late Miss Mawdesley has left £300, which comes to this year's balance-sheet. At the annual meeting appeals were made on behalf of the District Nursing which the Institution has taken up, and Deaconess Violet Hyde was congratulated at the way in which she had taken up, enlarged, and invigorated the work generally.

The first annual meeting of the **Denton Holme District Nursing Association**, held at Carlisle, showed that Nurse Keag's work had been appreciated, the total number of visits standing at 2,416. One drawback to the winter's work has been the illness of Nurse Keag, the tending of the sick having been stopped for nearly two months. Arrangements have been made to prevent a recurrence of this, the Guardians of the Carlisle Union having consented to allow one of the trained nurses from Fusehill Hospital to come when needed as a temporary assistant on specified terms. The Committee are hoping that subscribers will be generous and enable them to employ a probationer as well from this autumn. Surely Carlisle will afford this.

**Bradford Nurses.**—Since the Bradford Fever Hospital has been under Corporation management, the facilities for the training of the probationers of the Bradford Institution have been withdrawn, and this necessitates this branch of the training being carried on at Leeds. As the Mayor is lending his aid, it is hoped that this short-sighted policy on the part of the Corporation will soon be altered. The year's work seems to be well up to the average. This Institution sends out private nurses at reduced rates to those unable to pay the full charge, and does district nursing as well. There are plenty of rich men in Bradford and its suburbs, but, if we may judge by the balance-sheet of the nursing department, they seem to have heard very little of the fact that their poorer neighbours appreciate nursing in sickness.

**St. Alban's Diocesan Nursing Institution.**—At the annual meeting of the St. Alban's Diocesan Nursing Institution, held at Chelmsford, "the sincere and grateful thanks" of the meeting were put on record to the Hon. Mrs. Claughton for her wise direction of the affairs of the institution, and to Miss Mary Ann Luard, who, through ill-health, is resigning the post of Lady Superintendent, for her unwearied labours and indefatigable zeal. The nurses, averaging 28, spent 1,150 weeks in private houses, unions, and hospitals, and of these 290 weeks were spent in the houses of persons to whom the boon of reduced fees was granted, and 37 weeks in cottages where no fees could be paid. Altogether an excellent year's work was shown. The nursing department and the Walton Convalescent Home, which has hitherto been kept separately, will now be united.

**Bravo Torquay!**—The Committee of the Nurses' Institution have joined the National Pension Fund, on behalf of the nurses, and they will be grateful for more help to enable them to assist further in this satisfactory manner those nurses who, after many years of faithful service in the Institution, leave through increasing years or sickness. The Torquay Institution grows in work every year, and the nurses, now 23 in number, have moved to their new dwelling, 1 and 2, Woodville, Abbey Road. The number of visits paid in the year were 8,662. The district work is not generally known as widely as it should be, but it goes on steadily and quietly doing an immense amount of good, and the nurses help to save many valuable lives, and meet with a great many recognitions of gratitude from their poorer patients. Torquay Institution is entirely dependent on voluntary subscriptions.

### What "The Register of Trained Nurses" Really is.

Mr. Bonham Carter, Secretary of the Nightingale Fund, has sent us a pamphlet on the Registration of Nurses and the R.B.N.A., from which we take the following:—

The Royal British Nurses' Association has recently published a "Register of Trained Nurses for 1891," containing about 1,700 names, including midwives, and described as "the first fruits of three years' incessant work and organisation in this direction." "Mistakes," they say, "may possibly have been made, but no care has been spared to reduce the chances of error to a minimum." Let us note a few of the inaccuracies which have nevertheless been admitted. These examples relate to some of the best known hospitals, both in London and in the country.

Six nurses are registered as having belonged to one large hospital, of whom that hospital can produce no record. Another was there, but only for a month; and another was discharged as incompetent.

Eight of those who are registered as belonging to another London hospital failed to complete their training there, and of these, one was dismissed for dishonesty. Seventeen of the others were probationers only for short periods, such as three to six months. One of the persons registered as a certified nurse of the hospital was discharged for unkindness to her patient.

Five of those ascribed to a third London hospital had neither been trained nor certified there.

Of three persons registered as belonging to another hospital, one did come for a year's training, but was found unsuitable, and withdrew; another is credited with a certificate which she did not receive; and a third, also credited with a certificate, had never been in the hospital at all.

In yet another hospital one person, described by the register as holding its certificate, served only five months, and, therefore, could not possibly have been certified; two others were indifferent; two were unsatisfactory; and one was actually dismissed. Out of the whole number registered in this hospital one-half would not have been recommended for employment by their training school.

In another instance three persons are registered as belonging to a hospital where no record of them can be found. Therefore, if ever they were there, they must have left before the end of their month's training as unsatisfactory, and so were never put upon the books.

A second edition of this register has been published for 1892, in which, with a few exceptions, the whole of these errors have been repeated. So misleading may a register prove.

### To Relieve the Surplus Nursing Energy.

We quote the following from the *New York Medical Record*; part of the statement will strike our readers as a little wide of the mark: "The Wesleyan Church in England is about to establish a hospital of its own, the alleged reason for doing so being to give the female members of the sect an opportunity to nurse the sick. They say that they are now denied the privilege in the hospitals under the religious control of the Established Church. That's a grand idea, and we hope the Wesleyan doctors also approve."

### Nurses for the English in India.

We give below in full a letter from Mrs. Cuthell, the author of "Wanted Sick Nurses for India," and whom we mentioned in our pages a month ago. The subject demands consideration, fresh fields are becoming more and more difficult to discover, and yet here is a demand and no supply to satisfy it. Our article mentioned by Mrs. Cuthell was entitled "Fresh Fields," and was given in our issue of March 26th. Above all let it be remembered that such an enterprise in order to succeed needs thoroughly educated women whose training and diplomas must be of the highest standard.

*To the Editor of the Times.*

Sir,—While every year the Registrar-General's returns bring home to us the increasing number of surplus women as compared with men, yet employment for women is more and more difficult to obtain.

Allow me to call attention to the important opening there is in India for nursing among our own country-people and to the prospect to any certificated and efficient nurse of making a very good livelihood. A year ago I wrote on the subject in the *National Review*, and my views have since been corroborated in the same review by the wife of an Indian civlian of long standing, and also in the nurses organ, The Hospital for last month.

In England we utterly fail to realise the awful suddenness with which disease and death swoop down upon even the comparatively healthy in India. The fever fiend, the cholera king, ever hovering round, strike down here and there with merciless aim. Yet among the Anglo-Indians the aged are practically unknown, and there are few children over four or five, but the cemeteries are full of the graves of strong men, and the infants' corner is crowded. Nor is it only the climate that makes havoc among our country-people. Every one drives or rides; the animals are badly broken, often vicious; the equestrians and those who drive often inexperienced, and accidents are frequent. Polo, pig-sticking, racing, swell the list of catastrophes. The brick-like hardness of the sun-baked Indian soil renders a fall, which would hardly shake a man on our spongy turf, serious, and often fatal. Then there are numbers of civilians of all classes, many in isolated stations or lonely plantations, beyond the reach of a doctor, or whose duty takes them and their families out into camp for months together, moving from place to place, entirely cut off from the European population.

The Government system of civil and military surgeons and their subordinates, apothecaries, is excellent, and there is no need for private practitioners or consulting physicians, who are only to be found in the Presidency towns. It is not the heads, but the hands, the nurses, who are so urgently required, especially among the civilian class. The military are now well cared for by the Netley sisters, and, thanks to Lady Dufferin's efforts, the native women are not forgotten in their Zenanas. Even the leper on the highway is remembered. But civilians of all ranks are still dependent on a totally inadequate and precarious private supply of nurses. In the Presidency towns nursing sisterhoods do exist, but take a place like Allahabad, for instance, the seat of government of the North-West Provinces. The nursing there is, or was till recently, done by a few sergeants' wives, mostly Eurasians. There were but four certificated monthly nurses, and, these having to suffice also for the smaller out-stations, they are generally engaged a year deep, and are quite unavailable in cases of sudden illness. Sickness and sorrow bring out the best points in the somewhat frivolous Anglo-Indian feminine character. Devoted amateur nurses are often forthcoming; but, alas! how they blunder, how inexperienced they are!

Once in India, a qualified nurse would make a very comfortable livelihood; the pay is excellent, the demand large. Lady nurses would be well adapted to family life in India, for the houses are not arranged for European servants.

A writer in last month's Hospital suggests "that a group of thoroughly trained nurses should form themselves into a co-operation and go over to India to start a private nursing institution. There ought to be no difficulty in getting together a committee of medical men and ladies who have a knowledge of Indian life, and also in getting a lady superintendent to take the responsible post of head of the adventurous little band. It is no light thing to take up fresh work in a far country; it is only suitable for strong, well-trained women in the prime of life, who add to their love of nursing a spirit of adventure, and who can cheerfully risk some little capital in the hope of success in the new sphere."—I am, sir, yours faithfully, Edith E. Cuthell.

April 13th.

### The Nurses' Bookshelf.

### OPHTHALMIC NOTES.*

A modest little preface introduces us to a modest little book, if we judge only by the dimensions of the volume, but if we turn over the leaves we find ourselves passing from disease to disease, from accident to accident, until we sigh as we realise the number of mishaps to which one of the most necessary and most beautiful of our organs is liable. The author appears to have an honourable consciousness of the advantages he has derived from other writers and teachers on the subject of the eye, and he does not fail in mentioning the authorities whose practice he has from time to time adopted. He thinks, rightly, that his little volume will be of service to the advanced student, and he seems to have made it a very exhaustive pocket companion for anyone who is ambitious of mastering, in an easy manner, many most important details of modern treatment. The description of symptoms especially commends itself to us—such as the few lines which deal with sarcoma of the choroid (page 34), also the carefully-detailed explanations of operations for cataract—and the chapters dealing with "test-type" and ophthalmoscopic examination are valuable and comprehensive. That the writer is conversant with the practical as well as the theoretical side of his subject is evinced by his closing words: "The edge of cutting tools should be examined with a strong convex lens to detect dirt or imperfections." An appendix of remedies and an index are also parts of this admirable little book.

### HOW TO FEED THE BABY.†

Of the many admirable health tracts issued by the Ladies' Sanitary Association, that modest little society which has proved such a faithful pioneer to much hygiene work by women of all classes, the last which has come under our notice is perhaps one of the best of the series. An attractive blue binding encloses a great deal of useful information, which is conveyed in vigorous English, and in a plain and simple fashion which will recommend it to all nurses, as well as to the mothers, whose attention we specially call to this subject. Not only is the food for the baby dealt with in a thorough manner, but the proper and sufficient nourishment of the nursing mother herself receives particular notice, which is not always, or, indeed, often, accorded to this important subject. As to "weaning," and also as to the different kinds of milk, the boiling thereof, its legitimate and its improper dilution, and various other details, the advice is so exhaustive and so sound that the book will serve for useful reference for many a trained nurse, as well as for all probationers.

* "Ophthalmic Notes." By A. Vernon Ford, M.R.C.S.Eng., L.R.Q.C.P.Ire., &c., &c. (Published by Baillière, Tindall, and Cox.)

† "How to Feed the Baby." By J. Sinclair Holden, M.D. Issued by the Ladies' Sanitary Association, 22, Berners Street. Price 6d.

### Appointments.

[It is requested that successful candidates will send a copy of their applications and testimonials, with date of election, to The Editor, The Lodge, Porchester Square, W.]

Miss Kate Jones, who was trained at the London Hospital, has obtained the appointment of Night Superintendent at the Sussex County Hospital.

Charing Cross Hospital.—"Sister Margaret," who has held the appointment of Night Sister at this hospital, has now been appointed Sister-in-charge of Golding ward.

Miss Luard (niece to Miss M. A. Luard) will, in future, be financial Secretary and Superintendent of the Walton-on-the-Naze Convalescent Home, and Miss Montefiore the Matron of the Nurses' Home at Witham.

Miss M. I. Ratliff has been appointed Matron of the West Ham Hospital.

### On Manners.

This is a subject which concerns us all. Now that nurses take such an important position in public estimation, their actions, as well as themselves, are apt to sustain keen as well as appreciative criticism.

When nursing first began to attract the attention of educated women, many a modest girl's heart beat high with a hope that she might one day be a humble follower of the great pioneer, Miss Nightingale; but it was only one here and there who obtained the consent of her parents or guardians to enter on hospital life, and even if she made the attempt she still needed great courage to carry her past the obstacles which in those days beset her path. Still, when any lady did succeed in entering on, and keeping steadfastly to, a nursing career, she soon found herself adorned by her personal friends with a very halo of all the virtues, and possibly she may have merited some praise, although not nearly so much as she received, for she probably took up the work entirely from love of it.

Many of these first lady nurses had very little actual "training," their education and refinement enabled them to maintain a position considerably above the tribe of "Gamp," which flourished then; but their knowledge of the details which constitute skilled nursing, was only acquired by slow and laborious degrees. Owing their supremacy in the wards almost wholly to personal qualities, these ladies were usually possessed of good manners, in other words, of sympathy and kindly consideration for others. Of course this was not invariably the case, for we can, most of us, call to mind unfortunate examples of women, otherwise excellent characters in the nursing world, whose manners not the wildest flight of imagination could designate good. Yet, on the whole, the tone of those early days was one of gracious dignity. As time passed on, with the demand for highly-trained workers, so did the supply increase, until the last six or eight years have sufficed to flood the world with a noble army of nurses, some of them fitted by experience as well as knowledge to be most valuable assistants to the doctors, and to contribute not a little to the brilliant successes of famous surgeons by their skilful after-care of operation cases. With all this increase of knowledge and that perfect system of training which has rightly made our best British nursing schools of world-wide renown, dare we assert that yet everything is complete, whilst the manners of nurses still stand in need of reform? There are certainly one or two of our big hospitals where—all honour to those who have done it—courtesy to strangers is exacted as a duty from nurses and probationers, and we must trust that such politeness is not put on with the clean aprons, but that it becomes a characteristic of the wearers at all times and in all seasons, for it is a poor kind of courtesy which only flourishes in the presence of strangers, and is laid aside as superfluous for daily and hourly companions. We sigh, however, when we think of those hospitals where no sort of good manners prevail, where the stranger enters, ungreeted and unnoticed, and the newest probationer is less *gauche* than the nurse of ten years' experience. Is it vanity which makes these good women exhibit behaviour which would disgrace a factory girl? In the factory the girl may giggle or ignore the presence of a visitor, but in her home, poor though it be, she seldom fails to offer a greeting as well as a seat to the stranger who pays her the attention of a call. A ward is, in a certain sense, the nurse's home, inasmuch as it is under her charge, and is the place where most part of her time is spent, and she loves it, spares no pains in her care of it, and is devoted to the interests of her patients—but even to the latter her manner does not always do justice to the goodness of her heart; however, it is for her fellow nurses and for the unfortunate stranger that her worst manner is exhibited and we have sought, far and near, hitherto in vain, for an explanation of this hard fact. We do not find the failing confined to one class of women, and indeed we all know that many a cottager is as truly polite as a countess, and the plainly-reared nurse, whose very blunt honesty endears her to us, is often no ruder than the woman of higher birth. Why should either of these useful and honoured persons fall short in her duty, and why should the bad manners of some of our nurses be getting proverbial? If it is the outcome of indifference on their part, let there be an end of such folly; if it is the overweening importance they attach to their own useful career, let the unworthy thought die; the very pettiness of it detracts from the grace of their noble work. Bad manners may be defined as an ugly and visible form of selfishness. Thoughtful consideration for the feelings of all those with whom we are brought in contact ensures good manners, and when these are present, we find the idol of self-esteem has been very successfully relegated to a distant background. Many a good woman thinks the explanation, "It's only my way!" excuses that way for being an ungracious one; but far from it, out of her own mouth she is convicted, for her excuse is her accusation.

### Everybody's Opinion.

*[Correspondence on all subjects is invited, but we cannot in any way be responsible for the opinions expressed by our correspondents. No communications can be entertained if the name and address of the correspondent is not given, or unless one side of the paper only be written on.]*

### "TALKING SHOP."

"E. M." writes: I cannot help agreeing a little with "Anti-Shop" that nurses do a little too much talking about their professional duties. Of course there is much knowledge and information to be gained by discussion with our fellow-workers; but ideas certainly get narrowed if we do not let our thoughts and conversations dwell sometimes on other phases of life than ours. This is one of the many reasons why nurses should make a point of going out when they are off duty and seeing and hearing what is being done and said around them. We have all of us heard of the nurse who regales her patient with details of former cases, or who dilates on nursing generally to an exasperating extent; but there is no doubt she exists in fact, not in fiction, though the numbers of her rank are, we may hope, small. After all, who makes the better nurse, she who comes back from her outing full of fresh ideas and interests wherewith to distract her patient's mind from his all-absorbing ailment, or the nurse whose only topic is ailments? It is splendid to have found one's "life's work," but it is good to study that of others as well.

### HOLIDAY EXPERIENCES.

"Alpha" writes: I am sure there are many nurses who will agree with the writer of the article in The Hospital who said that nurses require variety in their holiday outings. Nothing so entirely changes one's thoughts as the novel experience of foreign travel. But there are so many who think that this is so surrounded with difficulties that they fail to make the attempt, and thus lose a great deal of pleasure. I have been very fortunate in my trips abroad, and I am sure that those who, like myself, once try the experiment, will repeat it often. I do not wish to make my letter too long, so will only give one holiday suggestion now, and if any nurse would like further particulars I shall be very happy to supply them. I imagine that ten days or a fortnight are to be devoted to the excursion, and I propose mine to go from London to St. Malo, in Brittany, as this is very easy to accomplish, and inexpensive too. You can go from London for about £2 return fare. The boats leave three times a week, and all particulars can be had from the agent, Mr. T. MacGarey, Arthur Street, E.C., who will send a small time table with all information. The voyage lasts nine hours, and the boats are fairly comfortable. From St. Malo there are charming excursions, and if nurses only take such luggage as a Gladstone bag, the pleasantest way would be to board by the day at one of the hotels or pensions, at about 6s. the day, and spend half the time at St. Malo, St. Servan, and Dinard, all quite close together, and the rest of the time at Dinan, twelve miles inland, proceeding there by river, which is very beautiful, and steamers run several times a week. Dinan is one of the most picturesque of all the old French towns, but I will not usurp the function of the guide book by saying more here. Those who have more money and time to spend should go and see the wonderful Mont St. Michael, which is quite a town within monastic walls. It is easily reached from St. Malo. Two holidays would not exhaust all the charming excursions to be made from this point, and the novelty of the scenery and quaint costumes of the people will give as pleasant and complete a change as any nurse could wish.

### Nurses' Homes.

### MARYLEBONE INFIRMARY.

It is somewhat of a surprise, on planning a visit to this institution, to find that it is situated at Notting Hill, on the high ground which overlooks Wormwood Scrubbs. However, the long journey is amply repaid by the first sight of the noble pile of buildings, where some 750 sick people are cared for, and opposite to which, though still within the gates, stands the home where the probationers live; that is to say, they sleep and spend their recreation time there, but all meals are served in the administrative block. The nursing home, which was built some seven years ago, is of grey stone, and has a very long picturesque front and plenty of unenclosed land around. In fact, open spaces and currents of fresh air seem distinctive features of the place and, bearing in mind the English climate, we are glad to find the probationers have a satisfactory heating arrangement of hot-water pipes in their home.

The Sister-Superintendent's sitting-room is conveniently placed near the entrance hall, and the large room where the nurses are free to pass their off-duty time, and in which the classes for instruction to the probationers are given three times a-week.

The Medical Officer gives lectures which appear to be highly appreciated, and the very beautiful "model" as well as the splints, bandages, prepared specimens of various "organs," &c., &c., all arranged in orderly array in suitable cupboards, give most satisfactory evidence of the thoroughness of the system of teaching.

We venture to assert that when all this paraphernalia is seen in good condition and free from dust, it is probably in frequent requisition, which is more than we dare say or think when a cupboard of fine "specimens" is pointed out to us, and we note the dirty accumulations on the glasses and the stiffness of a seldom used lock.

Just beyond this spacious and well-furnished sitting-room a staircase leads to the bed rooms of the nurses on "day duty," and the night nurses are quartered in the opposite wing, with a thick baize door to sh[illegible]t off all sounds. The little bed-rooms are comfortably furnished and bright-looking, with their white, scrubbed floors and fireplaces, though the latter are more for ventilation than as a means of warmth, for the heating apparatus already referred to is found sufficient, unless special circumstances render a fire necessary.

There is a charming bath-room to every five nurses, and the other sanitary arrangements appear to be equally good, the turret system being satisfactorily adopted. There are two cosy "invalid's rooms" leading into each other, and furnished respectively as bed and sitting room, and here the sick probationer is nursed back to health with a day and a night "special nurse" when necessary. In case of illness the "staff" or "charge" nurses are cared for in small rooms off their wards, and their ordinary bed-rooms are situated in the main building, for not only nurses and probationers, but every servant of the infirmary is accommodated with a separate sleeping room.

The Sisters, the charge nurses, and the probationers, have their meals served in three capital apartments, and, of course, at different hours. There is altogether something very home-like and comfortable, and pleasantly free from any of the bareness which we are apt to associate with the dining halls of schools and institutions in general.

Of the food, we hear that it is plain, good, and sufficient, and also that green vegetables, salad, and fruit, according to the season, form part of the diets; also eggs for tea seem a sensible provision.

The monthly day off duty to which each member of the nursing staff is entitled, is here made specially agreeable by the privilege, dear to all hospital workers, of breakfasting in bed, and thus a real holiday is secured, entirely free from responsibilities, and from a preliminary two or three hours of ward work which, in the busy early morning, are calculated to detract very much from the freshness of the probationer on her nominal "day" of freedom.

The Matron's house is conveniently placed on the right side of the courtyard or entrance, and is almost exactly midway between the main building and the nursing home. The office in which she transacts the business affairs of her large household is a fine, light room, in most perfect order.

There are two Night Superintendents, each one being responsible for half the infirmary, and they use for their head-quarters during the night the room where the day Sisters take their meals, and which, by the way, is not only a particularly pleasant, but also a conveniently central spot. The staff nurses have a sitting-room to themselves, and a piano, plenty of easy chairs, and other nice-looking furniture, all giving us the impression that the bodily rest of the workers is wisely provided for, as well as the cultivation of the brains and the training of the hands in this huge infirmary. The grounds surrounding and intervening between the blocks of building are specially to be commended, and we may hope that no more hideous factory chimneys will be permitted to encroach on the space and air at present dedicated to the service of these sick-poor and those who minister to them.

### Notes and Queries.

### Queries.

Will any home in or near London receive a woman of 69, partially paralysed, on payment of 4s. weekly?—*Sister, Nurses' Home, Plaistow, E.*

Will any district nurse inform me what composes a trousseau for district nursing? and what a district nursing home should possess?—*Sister F.*

### Answers.

*Nurse Margaret.*—Palpitation and heart failure caused by excessive smoking.

*C. C. P.*—You can get the best photograph ever taken of her at The Hospital Office, 140, Strand.

*Sister Barbara.*—You cannot do better than get the St. John's Ambulance pamphlets and books. Write to the Secretary, St. John's Gate, Clerkenwell, E.C., and tell him what you want them for.

*Coldstream.*—Your enquiry would necessitate a lengthy answer. You would find fullest particulars in the "Englishwoman's Year Book," published by Hatchard's, Piccadilly, One Shilling. There is the London School of Medicine for Women, Edinburgh ditto, and Dublin; these are the only recognised schools in this country. Nowhere else can you obtain complete education.

*C. M.*—Twenty-five guineas at Queen Charlotte's Lying-in Hospital, Marylebone Road, for three months; twenty guineas City of London Lying-in Hospital, for twelve weeks. Your best plan will be to write to Mrs. Nichol, the Secretary of the Midwives' Institute, 12, Buckingham Street, Strand, and ask her to advise you.

*F. G. G.*—Thanks for paper, which we will use.

*M.. E. W.*—The address of the Secretary of the Guild of St. Luke is Mr. H. Thoresly Jones, M.R.C.S., 103, Sutherland Avenue, Maida Vale, W.

### A Simple Deduction.

On the very day that her father was buried by the parish, Loo, a tall, slight girl of fourteen, fell ill. She had held on bravely hitherto; now, when there was no further need for exertion strength forsook her. A poor neighbour came in to visit her, and found her lying on the bed which the dead man had occupied so recently, her cheeks and eyes burning with fever, her parched lips parted. It was a case for the hospital, and to the hospital Loo was taken. She uttered no protest, and was not even aware of her change of abode. For days, indeed, she showed no signs of consciousness; when she did, she saw a face bending over her. It was a thin, pale face, with kindly eyes, and the thick hair that flowed back from the broad forehead was almost white.

Loo's brain was not quite clear yet; she stretched out her thin arms and wound them about the man's neck.

"Father!" she cried, with a sob, "is that really you? I thought I was all alone, and that you was dead. Wasn't I a silly, father, and you standing there all the while. I guess I've been dreaming, ain't I?"

"You are dreaming now, my child," said a quiet voice that was not her father's. "I am the hospital doctor. I've tried hard to get you well again, and, thank God, I've succeeded."

He could not speak clearly because of the pressure of her clinging arms, but all at once she withdrew them with a long, low sigh.

"Yes," she said slowly, "I understand. What a fool I was to mistake any one for father; I've been a bit off my head, ain't I? And you've been trying to get me well again," she observed.

"Why, certainly."

"It wasn't very neighbourly," said the girl, in a feeble far-away voice. "I'd have been better dead; there ain't nobody for me to work for now."

"You will get strong, my dear, and then we will see about getting you a nice comfortable situation."

She shook her head.

"I couldn't wait on any but men folk," she said, showing signs of excitement. "I ain't been used to anything else. First there was two of 'em, father and Joe. Joe was my brother; he went off in a galloping consumption, then there was only father; he kept hearty for a while till his lungs gave in too; nobody nursed him but me though."

"I didn't want to get better," she said with decision, "but I guess you meant well. Thank you kindly, sir. It ain't your fault that it is. Thank you for nothing."

He smiled gently.

"Then you won't be sorry to see me to-morrow morning?"

"Sorry, no! I shall be real glad. You're a bit like father in the face; not quite so handsome, of course, but the same cut of a man."

He came again next day, and found her lying pale and listless, but her face brightened at sight of him, and she answered his questions civilly. This went on for more than a week; then she took courage and put a question on her own account. It was plain and to the purpose.

"Are you a married man?" she asked.

"Yes," he said quietly; "but my wife died ten years ago."

"Got any children?"

He shook his head sadly.

"Bless my soul!" cried Loo; "whoever looks after you, then?"

He could not keep from laughing, though he was tenderly anxious not to hurt her feelings.

"There ain't any occasion to laugh," said she; "men folks want a deal of looking after. I know that; they likes their dinners tasty, and the place kept clean, and the hearth tidy."

The tears stood in her eyes as she strove to disarm ridicule; they crept into her voice likewise, and made speech difficult.

"You are quite right, my dear," said the doctor; "I was wrong to smile, but I have three good servants, and they know their duties."

Three good servants! Loo held her breath. Presently she said, sharply:

"You could do with two if you had a girl of your own. Don't you ever want somebody to love?"

"Yes, Loo," he said, with a sigh, "there are times when I do want somebody to love. But God knows best."

The girl made no rejoinder. She had for Dr. Severn an unusual charm, and his eyes loved to rest upon her delicate face. Yet he regretted that she was pretty. It seemed to him to place fresh obstacles in her path, and to open up new dangers. The nurse, finding her teachable, took pains to correct her speech. In a few weeks there was a manifest improvement. This the doctor noticed, and again he trembled for her, because she could so readily acquire all that she was taught. His interest was not merely sentimental. It took a practical turn. He represented her case to a lady friend, and she arranged to employ her in her kitchen. Loo was drafted off for three weeks to a convalescent home in the country, and when there wrote a little grateful letter to the doctor, who sighed as he read it, and refrained from throwing it into the fire.

On the evening of the day upon which the girl was due at her situation, Dr. Severn sat in his study, wearied after many hours of anxious labour. A sense of comfort stole over him, it was heightened by the sound of the wind and rain outside. Timid fingers rapped upon his door.

"Come in!" he cried, with suppressed impatience.

She burst in upon him like a whirlwind, her soaked garments clung to her, her curling hair was wet with rain, the tears stood upon her cheeks.

"Loo!" he cried, bending forward in his chair and shading his dazzled eyes. "Can that be Loo?"

She flung her arms about him, and laid her head upon his shoulder; then, as if horrorstruck at her own boldness, sank at his feet, and lifted her face to his.

"Three grown women servants," she cried, "and you could do with two easy, or one maybe, if you had a girl of your own. And, oh! if you please, I am used to men folks and their ways. And—and—"

She paused for breath, and clasped her hands together.

"Oh! you *must* want somebody to love," she urged, "when you haven't a child of your own. And if you'd take me you shouldn't ever know any difference. I promise faithful."

He stretched out his strong arms, and she crept into them sobbing for joy.

"My poor child," he cried, "you shall have your wish; my little Loo, my daughter."

Loo has kept her word, he has never known any difference.

---

### Wants and Workers.

Would any one be so kind as to send old sailor suits, sun bonnets, chintz curtains, or an old perambulator, or go-cart, to a small and poor cottage home for sick children in the Isle of Wight. Anything gratefully acknowledged by the *Matron, The Melicent Home, Sandown, Isle of Wight.*

## Figures and Tables

**Figure f1:**